# A descriptive study of fratricide in tunisia

**DOI:** 10.1192/j.eurpsy.2021.1019

**Published:** 2021-08-13

**Authors:** A. Guermazi, S. Omri, R. Feki, N. Smaoui, M. Maalej Bouali, L. Zouari, J. Ben Thabet, N. Charfi, M. Maalej

**Affiliations:** Psychiatry C Department, Hedi chaker University hospital, sfax, Tunisia

**Keywords:** fratricide, violence, perpetrators of fratricide and sororicide

## Abstract

**Introduction:**

Violence between brothers and / or sisters is one of the most important forms of violence within families. To understand homicides between them, the hypothesis of rivalry has been put forward. But how is it really in reality?

**Objectives:**

To construct both the clinical and medicolegal profile of perpetrators of fratricide and sororicide.

**Methods:**

This is a retrospective study of 12 cases of fratricide, which were examined in the context of criminal psychiatric expertise in the psychiatry department of Hedi Chaker University Hospital in Sfax (Tunisia), between January 2002 and December 2018.

**Results:**

The mean age of offenders was 31.9 years; they were all male. Eight fratricide perpetrators were unmarried and had an irregular occupation. They had a psychiatric follow-up prior to homicide in 5 cases. Previous criminal records were noted in one third of the cases. Three perpetrators of fratricide were using psychoactive substances. History of violence against the victim was presented in 7/12 of cases, and the victim was younger than the perpetrator in 5 cases. Aggression was premeditated in 4 cases. The knife was the most used weapon (11/12). Seven offenders suffered from a major mental illness. The most common diagnosis was schizophrenia (6/12). The experts had concluded that 8 cases were in a state of insanity at the time of the offense.

**Conclusions:**

Our data indicates that fratricides are lack preparation and most often preceded by violence. It seems to be important to do other researches to assess psychopathology and assess risk factors for fratricide.

